# Transcriptional Analysis of The Adaptive Digestive System of The Migratory Locust in Response to Plant Defensive Protease Inhibitors

**DOI:** 10.1038/srep32460

**Published:** 2016-09-01

**Authors:** Jornt Spit, Michiel Holtof, Liesbet Badisco, Lucia Vergauwen, Elise Vogel, Dries Knapen, Jozef Vanden Broeck

**Affiliations:** 1 Department of Animal Physiology and Neurobiology, Zoological Institute KU Leuven, Naamsestraat 59, B-3000 Leuven, Belgium; 2 Systemic Physiological and Ecotoxicological Research (SPHERE), Department of Biology, University of Antwerp, Groenenborgerlaan 171, B-2020 Antwerpen, Belgium; 3 Zebrafishlab, Veterinary Physiology and Biochemistry, Department of Veterinary Sciences, University of Antwerp, Universiteitsplein 1, B-2610 Wilrijk, Belgium

## Abstract

Herbivorous insects evolved adaptive mechanisms to compensate for the presence of plant defensive protease inhibitors (PI) in their food. The underlying regulatory mechanisms of these compensatory responses remain largely elusive. In the current study, we investigated the initiation of this adaptive response in the migratory locust, *Locusta migratoria*, via microarray analysis of gut tissues. Four hours after dietary uptake of PIs, 114 and 150 transcripts were respectively found up- or downregulated. The results suggest a quick trade-off between compensating for potential loss of digestive activity on the one hand, and stress tolerance, defense, and structural integrity of the gut on the other hand. We additionally addressed the role of a group of related upregulated hexamerin-like proteins in the PI-induced response. Simultaneous knockdown of corresponding transcripts by means of RNA interference resulted in a reduced capacity of the locust nymphs to cope with the effects of PI. Moreover, since insect hexamerins have been shown to bind Juvenile Hormone (JH), we also investigated the effect of JH on the proteolytic digestion in *L. migratoria*. Our results indicate that JH has a stimulatory effect on the expression of three homologous chymotrypsin genes, while knocking down the JH receptor (methoprene tolerant) led to opposite effects.

Plants and insects have continuously interacted with each other in a co-evolutionary relationship. While plants can benefit from the pollinator activities of several insect species, herbivorous insects use their host plants as main feeding source. The insect midgut shows a high flexibility, which allows them to swiftly adapt to varying nutrient levels in their diet^1^. A remarkable case of this high flexibility is found in the insect’s adaptive mechanisms to malnutritious factors. The constant threat of severe damage by feeding insects has led to the evolution of a wide range of specific protective strategies in the plants, among which secondary metabolites, such as protease inhibitors (PI)^2^. These PI primarily act in the digestive tract of the insect by targeting proteolytic digestive enzymes, or interfere with the structural properties or protective functions of the gut^3^. Resulting amino acid deficiencies will eventually manifest themselves in a limitation of the insect’s growth, development, fecundity, and can ultimately lead to an increased mortality^3^. Since their discovery, PI have unquestionably received a lot of attention in agricultural sciences, considering their insecticidal potential^4^,^5^.

However, several important pest insects have evolved mechanisms to compensate for the antimetabolic effects of PI, with attenuation, or a complete lack of developmental defects as a result^5^. Over the past decades, the number of published studies describing these different adaptive strategies has grown. These can generally be divided into two major categories: increased expression of active proteases, outnumbering the PI in the gut lumen, and/or expression of PI-insensitive proteases, which are not a target of the ingested PI^4^,^5^. Both strategies have been observed in many different insect species when confronted with PI^6^,^7^,^8^,^9^,^10^,^11^. Nevertheless, while much of the current literature focuses on how insects counteract the presence of PI, the current understanding of the initiation and regulation of these mechanisms still remains surprisingly unclear. This comprehension, however, could prove to be essential for the development of more effective PI-based strategies for pest control.

The migratory locust, *Locusta migratoria*, is considered among the most important agricultural pest insects, endangering an enormous acreage of cultivated areas worldwide each year^12^. Furthermore, its relatively large size makes it a highly suitable insect model for physiological research. Initial proteolytic digestion in this species is mainly steered by trypsins and chymotrypsins, complemented with a small activity of cysteine proteases^13^. We previously have shown that *L. migratoria* possesses a clear PI-based compensatory response^13^. It responds to the dietary uptake of soybean Bowman Birk (SBBI) and soybean trypsin inhibitors (SBTI) by inducing the expression of serine proteases active in the digestive system, while no effect on larval development could be observed^13^. Interestingly, we observed increased expression of serine proteases as quickly as four hours after the ingestion of the inhibitors, demonstrating the high flexibility of this response^13^. Like in other insects, the underlying regulatory mechanisms of this adaptation are however still unknown. Up to now, only few studies have tried to identify broader transcriptional changes associated with this phenomenon^8^,^14^,^15^,^16^,^17^,^18^. However, these studies mostly investigated long-term changes after dietary PI uptake and did not focus on the initial driving forces regulating the response.

Therefore, in this study, we conducted a microarray study to specifically observe short-term transcriptional changes in the gut and gastric caeca of the migratory locust at the previously observed critical time point of four hours after SBBI and SBTI ingestion^13^. Detailed analyses of differentially expressed transcripts showed a notable upregulation of several transcripts encoding hexamerin-like proteins, which are known to be juvenile hormone binding proteins (JHBPs)^19^. Interestingly, one other study performed in the Colorado potato beetle, *Leptinotarsa decemlineata*, also demonstrated an induced expression of several JHBPs after feeding on potato plants with induced defences^16^. These findings could suggest a more general role for JHBPs or JH in the regulation of the digestive process in insects. We therefore further pursued this idea. Knockdown studies by means of RNAi helped us reveal, for the first time, a possible regulatory mechanism involved in the PI-induced defence in this locust species. In addition, we were able to demonstrate a stimulatory effect of JH signalling on a specific group of serine proteases, further hinting towards a conserved role for this hormone in the regulation of digestion.

## Results and Discussion

### Two hundred and sixty-four differentially expressed transcripts were identified 4h after SBBI and SBTI ingestion

**Figure 1:**
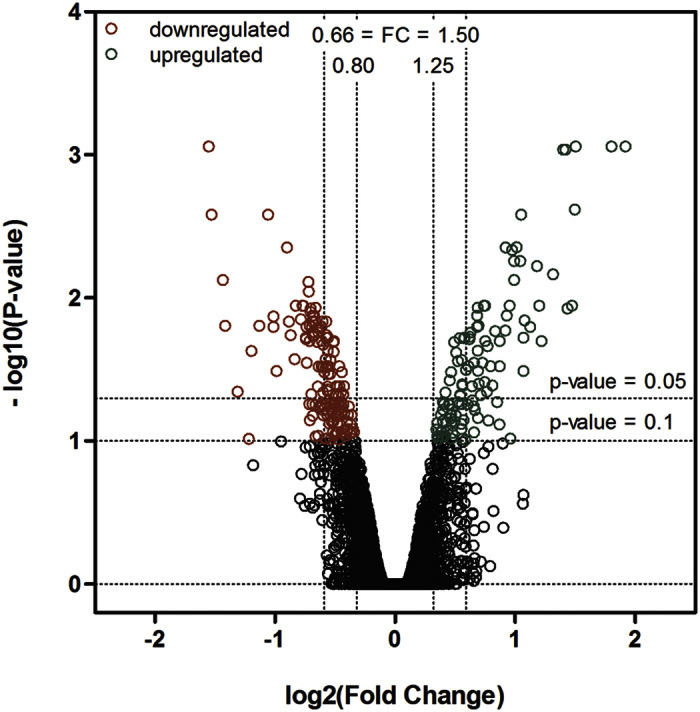
Volcano plot of the retrieved microarray data, plotting the negative log^10^ of the adjusted P-value against the log^2^ of the fold change (FC). This means that all transcripts with a FC < 1 have negative X-values and all transcripts with positive X-values have a FC > 1. The higher on the Y-axis, the lower the corresponding adjusted P-value. All values with a FC < 0.8, and P-value < 0.1 are indicated in red and are considered significantly down-regulated. All values with a FC > 1.25 and P-value < 0.1 are indicated in green and are considered upregulated.

Microarray slides were developed containing 35869 unique single probed transcripts (Spit *et al.*^20^). Transcript levels of combined midgut and caeca samples originating from 5th instar nymphs, 4 hours after exposure to a diet containing SBBI and SBTI (1%), were compared to those from control locusts. These plant derived inhibitors are well described and capable of inhibiting both insect trypsins and chymotrypsins, which constitute up to 90% of the total proteolytic activity in the digestive system of *L. migratoria*^21^,^22^,^23^. The administered dose of 1% of total protein content was previously shown to be successful in eliciting a quick response in the locust gut^13^. Figure 1 shows a volcano plot representing the results. Plotting the negative log10 function of the adjusted P-value against the log2 function of the fold change, visualises the up- and downregulated transcripts as a response to PI uptake. All transcripts with increasing or decreasing fold changes of at least 25% at an adjusted P-value cut-off of p < 0.1 were considered differentially expressed as result of the treatment. In total, 114 and 150 transcripts were identified as respectively up- or down-regulated. A higher number of downregulated genes is not surprising, as it has been previously noticed that the expression of several genes is suppressed rather than induced under environmental stress^24^.

To confirm expression results from the microarray, several transcripts from the top right and top left corner of the volcano plot were selected, representing strongly up- or downregulated transcripts, respectively. In total, 7 transcripts were tested with RT-qPCR: LMC_002270, LMC_003930, LMC_000698, LMC_000083, LMC_003601, LMC_003606, and LMC_004513. Transcript levels were measured in a new group of 5^th^ larval locusts that were fed a diet containing SBBI and SBTI, and with the exception of LMC_000083, expression levels did not differ significantly from the fold changes as observed in the microarray analysis (Supp. Fig. S1).

### Functional annotation of SBBI- and SBTI-responsive genes from *L. migratoria*

**Figure 2:**
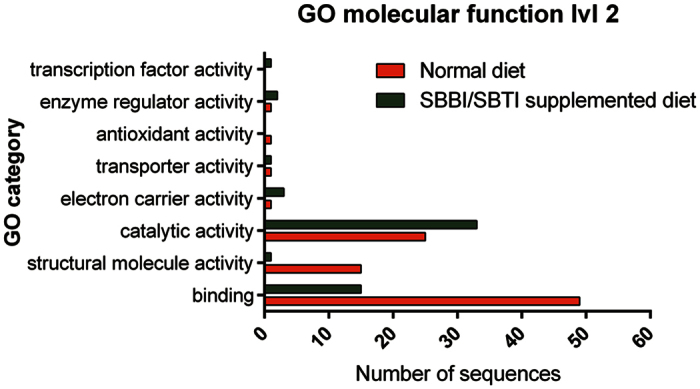
GO term classification of differentially expressed annotated transcripts under GO category ‘*Molecular function’* level2. Comparison of transcript numbers associated with different GO terms that are more abundant in control locusts (red) or locusts fed on a diet containing PI (green).

Sequences of differentially regulated transcripts were functionally annotated using gene ontology (GO), and the InterProScan (IPS) tool that is integrated in the Blast2GO bioinformatics platform^25^,^26^. Out of 264, 95 transcripts did not produce any significant BLAST hit. 113 of the remaining 169 sequences with at least one hit were ultimately assigned GO terms. Comparing the main GO ontology domain ‘Molecular function’ (lvl2) shows that most transcripts are putatively involved in ‘catalytic activity’, ‘binding’ or ‘structural molecule activity’, with the latter two more dominantly present in insects that received control diet (Fig. 2). Annotation numbers resulting from the IPS are comparable. 133 sequences could be assigned to an InterPro family. From these sequences, 68 belonged to transcripts that were upregulated, and 65 to transcripts that were downregulated. Tables 1 and 2 show a summary of the results for respectively the up- and the down-regulated transcripts. A list containing all up- and downregulated transcript IDs with their corresponding LogFC, calculated fold change, and putative sequence description is provided as supplementary data (Table S2).

#### SBBI and SBTI ingestion quickly induces changes in the digestive enzyme profile

Immediately apparent is the large number of upregulated sequences involved in protein digestion. Several transcripts are predicted S1 serine endoproteases, while others correspond to putative carboxypeptidases. Our data demonstrate that *L. migratoria* counteracts the malnutritious effects of SBBI and SBTI in the gut by inducing the expression of serine endoproteases, thereby supporting our previous research^13^. In addition, the observed elevated transcript levels for carboxypeptidases, suggest partial use of this class of exopeptidases to overcome the inhibitory effect of the ingested endoprotease inhibitors. This is the first time that this type of compensation is observed in *L. migratoria*. Partial reliance on insensitive proteases as counter defence however has already been observed in different other insects, including: *Helicoverpa armigera, Spodoptera exigua*, *Callosobruchus maculatus, Leptinotarsa decemlineata and Schistocerca gregaria*^9^,^14^,^16^,^27^,^28^. Our findings confirm that regulating the expression of proteolytic enzymes is a common strategy applied by insects to evade loss of proteolytic activity caused by the presence of PI in the gut. Surprisingly, in contrast to the observed trend of general upregulation of digestive proteases, one aminopeptidase and a dipeptidase sequence were found downregulated in response to dietary PI uptake (Table 2). The underlying reason for this is unclear. In some insects there exists a trade-off between the expression of proteases susceptible to the ingested PI and insensitive proteases^3^,^9^. However, the used protease inhibitors, SBBI and SBTI, are not capable of inhibiting aminopeptidases or dipeptidases. A possible explanation could be that the expression of these proteases is influenced by the levels of free amino acids present in the gut. When these levels are affected by the presence of PI, downregulation of these enzymes might occur.

The locusts’ exposure to PI also results in an upregulation of a group of enzymes involved in lipid metabolism and carbohydrate hydrolysis (Table 1). This could be consistent with an exploitation of alternative sources of energy to compensate for a loss in energy normally derived from protein digestion^14^,^18^. On the other hand, a β-glucosidase (encoded by 3 transcripts), two hexokinases and cellulase were found downregulated under the same conditions. Interestingly, the simultaneous downregulation of β-glucosidase and cellulase, while other carbohydrate hydrolases were upregulated, was also observed in microarray studies from *L. decemlineata*^16^, and *C. maculatu*s^14^. In addition, a low-density lipoprotein (LDL) receptor and an apo-lipoprotein (lipocalin) were also downregulated. Both are involved in the trafficking of lipids to energy demanding tissues or the fat body, suggesting less energy is available for redirecting or building reserves. Other enzymes including short- and long chain dehydrogenase, nucleoside hydrolase and carbonic anhydrase were also downregulated, indicating a metabolic adjustment to use energy for other tasks, like the observed enhanced proteolytic digestion.

#### Structural integrity and defences are in a trade-off with compensatory expression of digestive enzymes

Another intriguing observation is that, after being confronted with SBBI and SBTI in their gut, locusts respond by suppression of transcripts encoding proteins involved in both muscle and cytoskeleton structure as well as cell adhesion (Table 2). This suggests a weakening of the structural integrity of the insect gut in response to PI uptake. However, 4 hours after PI ingestion, structural changes were not yet visible. It could certainly be of interest to further investigate if the muscle structure of the gut indeed is compromised at later stages. Changes in the ultrastructure of the midgut epithelium have been observed in longer term studies on the PI compensatory responses in insects. In the black field cricket, *Teleogryllus commodus*, reduction of midgut wall dept, vacuolization of epithelial cells, swelling of the microvilli and eventual rupture of epithelial cells was observed after consecutive days of ingesting PI^29^. In *Drosophila melanogaster,* a remarkable reduction in the length of midgut microvilli was also observed in SBBI-fed larvae^30^. Other studies investigating morphological changes in response to PI ingestion are lacking, but these results suggest that structural changes may indeed coincide with PI ingestion in insects.

Interestingly, reduced transcript levels for several defence- and stress response related genes are also observed (Table 2). Among these are transcripts encoding C-type lectin proteins, which mainly function in innate immunity as microbial pattern recognition molecules^31^, while haem peroxidases and cytochrome P450-like enzymes are generally known to be important during oxidative stress. Heat shock protein HSP20 was also found downregulated. In insects, HSP20 has been reported to play critical roles during thermal adaptation^32^,^33^. In addition, carboxylesterase encoding transcripts were downregulated, which were previously shown to be involved in the xenobiotic detoxification in *L. migratoria*^34^. Combined, these results indicate that, while executing the counter defence against the dietary challenge presented by PI, locusts may be more susceptible for the risks of other environmental factors. In contrast, another carboxylesterase B-like sequence, and some additional cytochrome P450 encoding transcripts were found upregulated, which could indicate a more specific role for these proteins during PI-compensation in the locust gut. While structural properties of the gut appear negatively affected, chitin binding mucin-like peritrophins were upregulated after PI ingestion. These proteins are part of the peritrophic membrane (PM) that surrounds the food bolus in insects, protecting the midgut epithelium from microorganisms and mechanical damage. This suggests a strengthening of the PM after PI detection in the gut. Similar results were obtained in corn rootworm larvae during scN inhibitor challenge^35^. It was suggested that a decrease in protein degradation efficiency could lead to a more abrasive food bolus, so a more rapid turnover of the PM is required to protect the midgut epithelium^35^.

#### Juvenile hormone binding proteins (JHBPs) are upregulated

Among the most highly upregulated transcripts is a large group of sequences encoding putative hexamerin-like proteins. Hexamerins are high molecular weight proteins comprised of six identical or similar subunits. They are related to hemocyanin, but lost their ability to bind oxygen. Hexamerins are best known as hemolymph storage proteins providing amino acids and energy during non-feeding periods^36^. More recently, additional functions for this protein family were suggested. It could be demonstrated that hexamerins are phase-dependently expressed in both *L. migratoria*^37^ and *S. gregaria*^38^. Moreover, two hexamerin genes were found to participate in the JH-dependent regulation of caste polyphenism in termites^39^, suggesting their importance in regulating phenotypic plasticity. Apart from their function as storage proteins, in *L. migratoria,* hexamerins were also reported as juvenile hormone binding proteins (JHBPs) that function in the regulation of JH levels^19^. Interestingly, a family of low molecular weight JHBPs was also found upregulated in a study of the response of *L. decemlineata* to induced potato plant defences^16^. These findings prompted us to perform a comprehensive search in the Supplementary Data of related microarray studies, revealing several genes involved in JH production and degradation, e.g. JH acid methyltransferase, JH esterase and JH epoxide hydrolase, were affected during PI compensatory responses in *C. maculatus* and *H. virescens*^14^,^15^. Combined, these results indicate that JH and/or JHBPs are potentially more directly involved in the regulation of digestion and the proteolytic compensation after dietary exposure to antinutritional components. This idea is further supported by a growing number of reports on JH regulating the expression of certain proteolytic enzymes in the midgut^40^,^41^,^42^,^44^.

### Hexamerin knockdown perturbs the PI compensatory response

The remarkable consensus between some of the results from our microarray study and those from other studies on insects fed with different kinds of PI could suggest that similar regulatory networks are present in different orders of insects. One of the most intriguing results from these studies is the differential expression of JHBP family members, suggesting an important role in the regulation of the PI-induced compensatory response.

**Figure 3:**
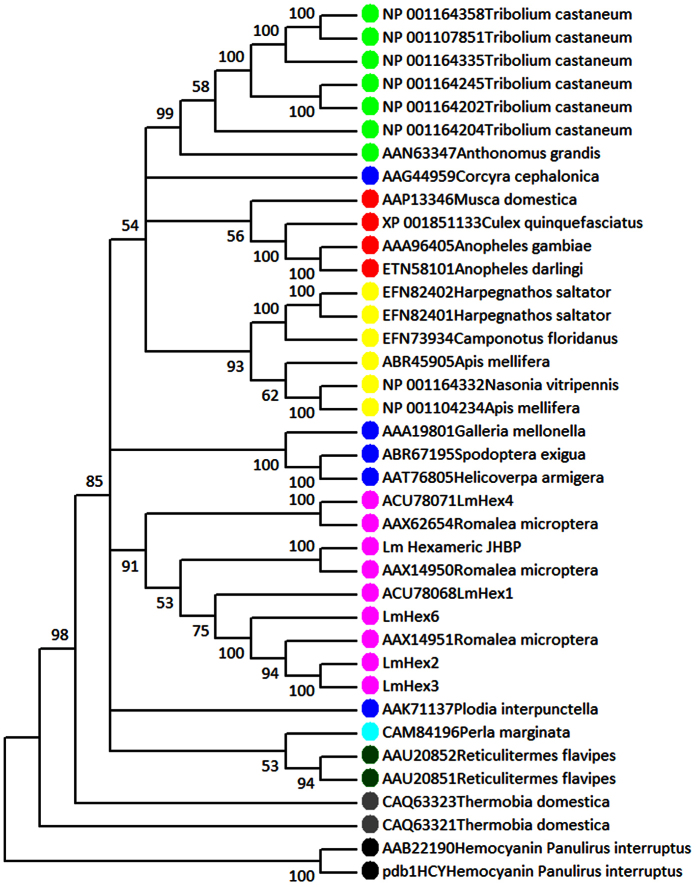
Maximum Likelihood tree of insect hexamerin-like proteins. Protein sequences of hemocyanin from *Panulirus interruptus* were used as outgroup to root the tree. The tree was inferred from 100 bootstrap replicates and bootstrap values for every node are presented. Accession numbers and species name are depicted. *S*equences from Orthoptera are represented in purple, Coleoptera in light green, Lepidoptera in dark blue, Diptera in red, Hymenoptera in yellow, Plecoptera in light blue, Isoptera in dark green, Zygentoma in grey.

The microarray analyses revealed a set of 21 differentially expressed transcripts encoding hexamerin-like proteins in the larval gut after ingestion of SBBI and SBTI. Many of these transcripts appeared closely related or identical to each other and showed substantial similarity (>98% identity). Based on sequence identity of (overlapping) nucleotide sequences, the transcripts could be further assembled into three distinct full length contig sequences. For all three contigs a coding region from start to stop codon was present, together with 5′ and 3′ UTRs (Supp. Fig. S3). For *L. migratoria*, five sequences encoding hexamerin-like proteins and one so-called hexameric JHBP can be found in GenBank. Contig 2 was identical to hexamerin-like protein 2 (*LmHex 2*; [GenBank: FJ609739.1]), while contig 3 was identical to hexamerin-like protein 3 (*LmHex 3*; [GenBank: FJ609740.1]). The sequence from contig 1 was not identical to any known hexamerin sequence from *L. migratoria* and was therefore designated as *L. migratoria* hexamerin-like protein 6 (*LmHex6*). Its sequence was confirmed by PCR and uploaded to Genbank (accession number: BK009413). Phylogenetic analysis shows that the hexamerin-like sequences from *Locusta* and other Orthoptera form a monophyletic group within the insect hexamerins, which was supported by high bootstrap values (Fig. 3). The sequence for LmHex5 was not included in this analysis since only a partial sequence for this protein was present in GenBank. The amino acid sequence of LmHex2 was most similar to LmHex3 (96%), while they both share 75% identity with LmHex6. The identity with, and between, other hexamerin-like proteins from *L. migratoria* varies between 35% and 45%, suggesting *LmHex2*, *LmHex3*, and *LmHex6* are highly related genes (Supp. Table S4).

**Figure 4:**
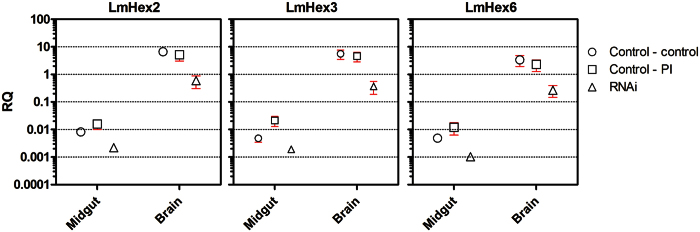
Mean relative transcript levels for hexamerin-like proteins after knockdown in combination with protease inhibitor feeding. Relative transcript levels in midguts and brains were examined for 5^th^ larval locusts that received dsRNA for gfp as a control and were subsequently fed a control diet (open circles), or a diet containing SBBI and SBTI (open squares), and were compared to locusts that received a combination of dsRNA for *LmHex2, 3* and *6* (open triangles). Hexamerin expression data for RNAi–control and RNAi–PI data were combined since no difference in transcript levels could be detected between these two groups. Relative transcript levels are normalized against two reference genes, *rp49* and *rps13*. Axes are scaled logarithmically, and each horizontal dotted line represents a 10 fold difference in expression. RQ = relative quantity. Means ± SEM are represented (n = 5 pools of >5 individuals). Statistical differences were determined by ANOVA. For both brain and midgut, transcript levels were significantly reduced by RNAi (p < 0.01).

In order to more closely evaluate the function of these hexamerin-like proteins, the transcript levels for *LmHex2*, *LmHex3*, and *LmHex6* were determined in 11 different adult locust tissues under normal dietary conditions (Supp. Fig. S5). Expression of all three was highest in the fat body, the flight muscles, the central nervous system and the gonads. High transcript levels in the gonads suggest that hexamerins may supply amino acids for gonad development and gamete production in *L. migratoria* adults, while expression in the flight muscles supports the idea that hexamerins could be used for flight activity^45^. Alternatively, expression of hexamerin-like proteins in these tissues might also be related to their JH binding capacity and influence the transport of JH in or out of these tissues, thereby regulating reproductive and developmental physiology. Surprisingly, in sharp contrast with the high expression level in these tissues, transcript levels of *LmHex2, LmHex3* and *LmHex6* were very low in the foregut, midgut, hindgut, gastric caeca and Malpighian tubules, which are all associated with the alimentary tract. However, when fed with PI rich diet, the transcript levels of hexamerins were altered in the gut. No clearly detectable changes could be observed in the brain (Fig. 4). These results could hint towards a more specified role of hexamerins in the gut during variable dietary conditions.

**Figure 5:**
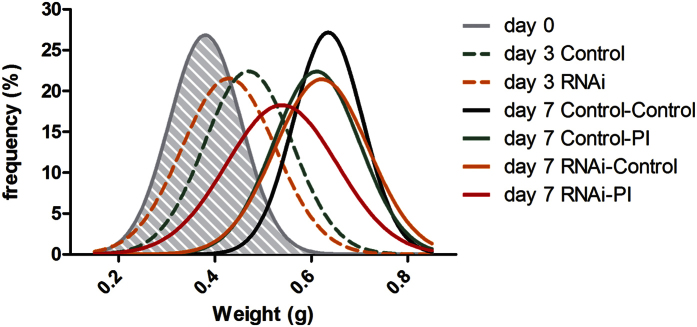
Frequency distribution of weight from control and experimental 5^th^ instar locust larvae at different time points. At day 0, half the locust population was injected with dsRNA for *LmHex2*, *LmHex3* and *LmHex6*, or dsRNA for gfp as a control. After three days half the locusts from each group received a diet containing SBBI and SBTI, while the other half received a control diet supplemented with BSA. Weight gain was recorded during the experiment and is presented as frequency distributions for each subsequent subpopulation. Each final group contained at least 30 individuals, both male and female. Statistical differences in weight (ANOVA) are further discussed in the text.

To further determine the possible role of these proteins in the flexible regulation of digestion, we performed a simultaneous knockdown of the three hexamerin-like sequences. Seven days after injection of dsRNA, the transcript levels of *LmHex2, LmHex3 and LmHex6* were reduced with respectively 95%, 93% and 94% in the locusts’ brains (Fig. 4). It must be noted that, while a very high knockdown efficiency was achieved, brain transcript levels after knockdown were still remarkably higher than the transcript levels in the gut of control animals. Nevertheless, while transcript levels in the gut were already low in control animals, they could still be significantly reduced through dsRNA injection (73%, 76% and 88%). To investigate the role of the knocked down genes in the PI-induced compensatory response, experimental locusts were fed with either a PI rich diet or a control diet. These conditions were compared with the same dietary conditions in control locusts. Individual locusts were weighed throughout the experiment. In this set-up, failure in the compensatory response would manifest in differences in weight gain. All groups significantly gained weight after three and seven days. Mean weight was significantly different after 3 days for control versus RNAi treated locusts (T-test; p < 0.05). Nevertheless, RNAi treated animals fed with a control diet regained their normal growth rate and at the end of the experiment their weight was no longer statistically different from control animals grown on a control or PI-rich diet (Fig. 5). In contrast, knockdown of two important storage proteins, SeHex and SeSp1 in *Spodoptera exigua* resulted in increased mortality, suggested to be caused by a lack of nutritional reserves^46^. However, in this study, we only targeted the group of hexamerins that were upregulated in the gut after PI ingestion. Additional storage proteins or hexamerins exist^19^, which could explain the lack of noticeable developmental defects in the PI-untreated knockdown condition. Mean weight of insects feeding for 4 days on PI-enriched diet also did not differ significantly from control animals, confirming swift adaptation of *L. migratoria* to SBBI and SBTI. Opposed to the other conditions, the growth of locusts that received *hexamerin*-dsRNA in combination with SBBI and SBTI slowed down, and at the end of the experiment the mean weight of this group was significantly lower compared to all other groups (ANOVA; p < 0.01).

**Figure 6:**
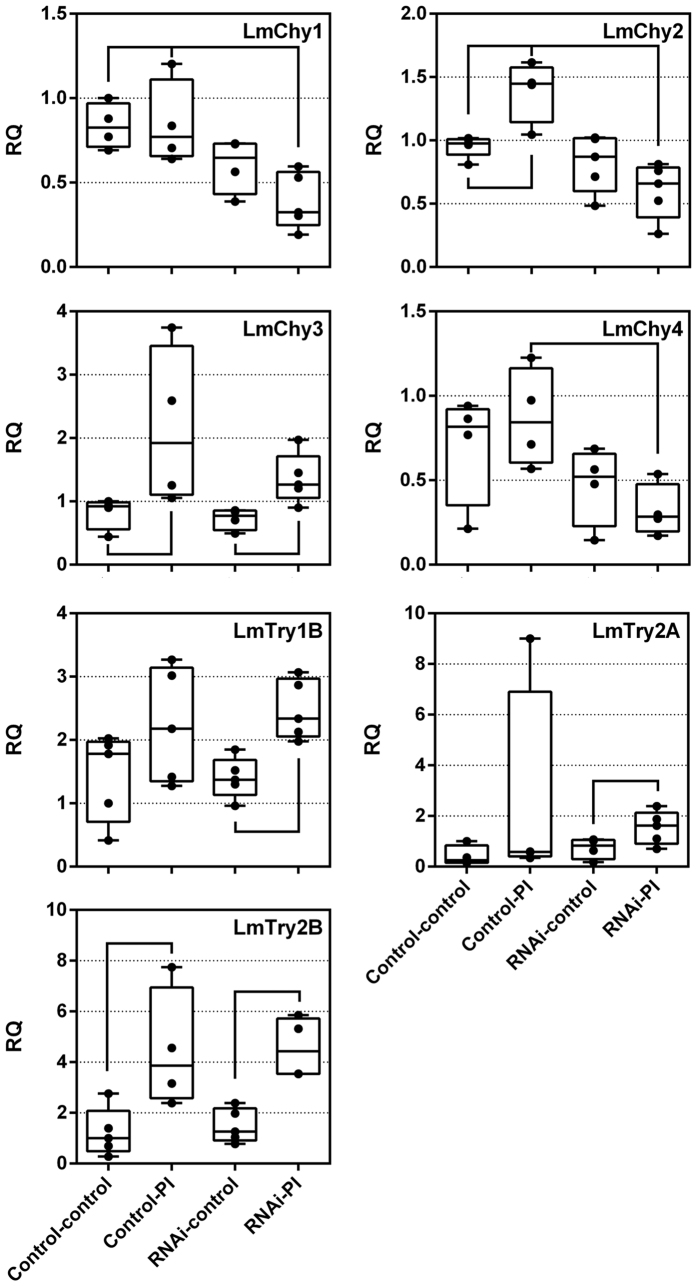
Relative transcript levels of selected proteases after knockdown of *LmHex2*, *LmHex3*, and *LmHex6*, plus PI ingestion. Transcript levels in the midgut were normalized against two reference genes, *rp49* and *rps13*. Box plots are presented based on five pools of over five individuals. RQ = relative quantity. Relevant statistically significant differences are visualized by connecting black lines (p < 0.05, one way ANOVA).

To understand the underlying reason for the observed delay in growth, the transcript levels of several serine protease genes that were previously shown to be upregulated in *L. migratoria* nymphs after prolonged exposure to SBBI and SBTI uptake were analyzed after day 7 (Fig. 6). With the exception of *LmChy1* [GenBank:BK008825], our results confirmed upregulation of serine proteases after PI ingestion, be it slightly less pronounced than previously reported^13^. Interestingly, transcript levels of *LmChy1, LmChy2* [GenBank:BK008826] and *LmChy4* [GenBank:BK008828] are lower in locusts that were additionally fed a diet containing PI (Fig. 6). Transcript levels of *LmChy3* [GenBank:BK008827]*, LmTry1B* [GenBank:BK008820]*, LmTry2A* [GenBank:BK008822] and *LmTry2B* [GenBank:BK008823] are not affected by the treatment, and the usual upregulation of these genes after PI ingestion is still observed after PI ingestion of RNAi animals. Remarkably, our previous work showed that *LmChy1, LmChy2* and *LmChy4* tightly cluster in a phylogenetic analysis of *L. migratoria* serine proteases^13^. This could indicate that their expression might be under the same regulatory control mechanism. Lowered expression of several proteases, normally contributing significantly to the proteolytic digestion, could help explain the reduced weight gain observed in RNAi animals fed with the PI-containing diet. Nonetheless, upregulation of some proteases was retained, suggesting that sufficient proteolysis remained to ensure the insects’ survival, albeit at a slightly slower growth rate.

### Juvenile hormone signalling has an effect on the expression of *LmChy1, LmChy2 and LmChy4*

The reported ability of hexamerins to bind and transport JH in the hemolymph^19^, and the effects of a knockdown on *LmChy1, LmChy2,* and *LmChy4* expression during a PI-induced compensatory response led us to further investigate the possible role of JH in the regulation of the proteolytic digestion. We approached this hypothesis in two different ways. First, by interfering with JH signalling, through the knockdown of the sole known JH receptor, methoprene tolerant (*LmMet*)^47^. Second, by stimulating JH signalling through topical application of methoprene, which acts as JH analogue.

**Figure 7:**
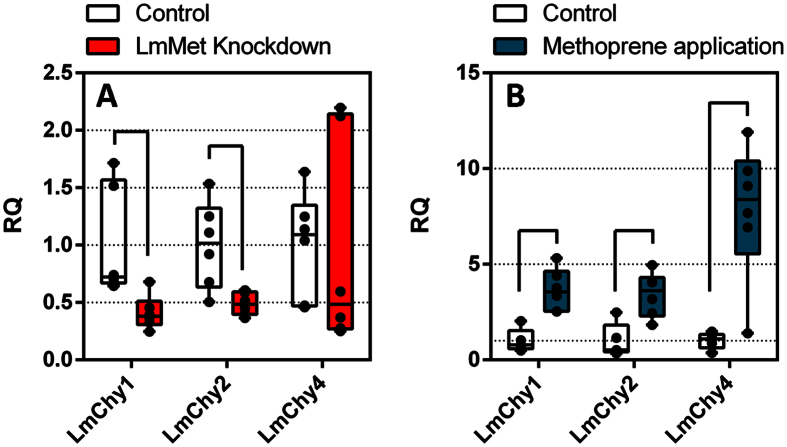
Relative transcript levels of *LmChy1, LmChy2* and *LmChy4* after *LmMet* knockdown (**A**) and after topical application of methoprene (**B**). Transcript levels in the midgut were normalized against two reference genes, *rp49* and *rps13.* Box plots are presented based on six pools of at least four individuals. RQ = relative quantity. Significant differences are visualized by connecting black lines (p < 0.05, MWW).

An RNAi-mediated knockdown of the *Met* gene [GenBank: KF471131.1] was performed using an identical dsRNA construct as reported by Guo *et al.*^48^. An average knockdown efficiency of 50% could be observed in the brain. However, reaching similar knockdown values in the midgut proved difficult (Supp. Fig. S6). Nevertheless, effects on protease expression were still clearly detectable. Figure 7A shows that the knockdown of *LmMet* results in significantly decreased transcript levels of the chymotrypsin genes, *LmChy1, LmChy2* (MWW; p < 0.01), while transcript levels of *LmChy4* show a strong trend in the same direction. Interestingly, this is the same group of sequences that was affected by the hexamerin knockdown. No changes in transcript levels of any other serine proteases were detected (Supp. Fig. S7). Treatment of locust nymphs with methoprene results in the opposite effect. As shown in Fig. 7B, the transcript levels *of LmChy1, LmChy2 and LmChy4* are clearly induced in the methoprene treated locusts compared to the control group (MWW; p < 0.01). Other serine proteases were again not affected by the treatment (Supp. Fig. S7). In addition, we also observed a significant stimulation in the expression of the hexamerin genes, *LmHex 2*, *LmHex 3, and LmHex 6* in the midgut, confirming the successful application of methoprene and subsequent stimulation of JH signalling in the insects (Supp. Fig. S7). The transcript levels of *LmMet* did not differ.

Juvenile hormone is one of the most studied insect hormones. It has several well-described functions throughout the lifespan of all insects, but it is best known for its role as regulator of larval moulting and reproductive maturation^49^. In female insects, the cross talk between JH and feeding, mainly through the insulin and Target of rapamycin (TOR) nutrient sensing pathways, is vital for the production of yolk proteins in the fat body. The underlying mechanisms of this interaction however still remain largely elusive^50^. Although these findings indicate a link between JH and feeding, the direct involvement of JH in the regulation of the digestive system hasn’t been studied in great detail yet. There exist scarce reports on certain digestive proteases seemingly under the regulatory control of JH^41^,^42^,^43^,^44^,^51^. However, the best-studied case of JH involvement in the digestive system of an insect species comes from the mosquito, *Aedes aegypti*. It is well established that the ingestion of a blood meal in the female mosquito takes place in two distinct phases. A first stage when a small amount of early trypsins initiates the proteolytic digestion is always followed by a second stage when much larger amounts of late trypsins continue this digestion. Studies have shown that the transcription of the early trypsins is under strict regulatory control of JH^52^. Based on feeding behaviour, insects can roughly be subdivided into two major categories. There are the non-continuous feeders (predators and hematophagous insects, such as mosquitoes), and the more continuous feeders (e.g. Coleoptera, Lepidoptera, Orthoptera). In general, it is believed that the digestive process in continuous feeders is more or less constitutive, whereas in discontinuous feeders, the digestive system is more tightly regulated^53^,^54^. Interestingly, phylogenetic analyses have shown a clear divergence of insect trypsins and chymotrypsins. Dipteran early trypsins appear to be more related to an ancient trypsin molecule, while late trypsins show much more sequence resemblance with insect chymotrypsins^13^,^55^. In addition, *LmChy1, LmChy2* and *LmChy4* also appear to have evolved from a trypsin ancestor^13^. The present data therefore indicate that the digestive system of continuous feeders, like locusts, could also be regulated up to a certain level by JH, and that this function of JH on certain digestive enzymes could be present in a much broader group of insects. This leads us to hypothesize an evolutionary conserved role of JH in the digestive system in insects, but has to be further investigated in more species.

## Conclusions

Exploiting natural plant defence mechanisms, such as antinutritional PI, is an attractive option to generate future insect resistant crops. Nevertheless, several pest species possess a remarkably flexible digestive system, allowing them to circumvent the presence of PI in their gut and avoid developmental defects. Our previous research demonstrated that an increase in proteolytic enzyme expression could already be observed four hours after the dietary uptake of SBBI and SBTi (1%) in *L. migratoria*. Here, we therefore investigated large scale transcriptional changes that occur in the midgut and gastric caeca of fifth instar *L. migratoria* nymphs, four hours after dietary uptake of SBBI and SBTI. In total, the levels of 264 transcripts were found to be altered in these conditions. The results indicate that fewer resources will be invested in immunity and structural integrity of the gut, to be able to compensate for the presence of the PI in the intestinal tract, through adjusting the digestive enzyme profile. Moreover, a group of related upregulated transcripts suggests hexamerin-like proteins likely fulfil an important role in the compensation process. The specific function of hexamerin-like proteins as JHBPs led us to further examine the possible role of hexamerins and JH in the regulation of proteolytic digestion in the migratory locust. Our combined results clearly suggest a role for both hexamerins and JH in the regulation of the expression of a specific set of proteolytic enzyme encoding genes in the migratory locust. Under regular dietary conditions, JH signalling (via the methoprene tolerant receptor) elicits a significant stimulatory effect on the expression of a specific group of chymotrypsin-like serine proteases: *LmChy1, LmChy2* and *LmChy4*. However, when their diet contains elevated levels of PI, more proteases are needed. One plausible strategy to meet these demands relies on a JH-stimulated overexpression of digestive proteases. In order to capture more JH in the digestive tract, hexamerins (or other JHBPs) may act as specific carriers of the hormone. Hence, we hypothesize that after PI uptake, transcript levels of hexamerins are swiftly upregulated in the gut to assist the JH-stimulated expression of certain proteases.

## Materials and Methods

### Rearing of animals and sample collection

Locusts (*Locusta migratoria*) were reared under crowded conditions with controlled temperature of 32 °C, light (14 h photoperiod) and relative humidity (40–60%). Locusts were fed daily with grass. For experiments, nymphs were developmentally synchronized on the day of the 5th larval molt (Day 0). For extraction of total RNA, tissues were dissected in *L. migratoria* Ringer’s solution (composition: 9.82 g/l NaCl; 0.48 g/l KCl; 0.19 g/l NaH2PO4; 0.25 g/l NaHCO3; 0.73 g/l MgCl2; 0.32 g/l CaCl2; pH 6.5) and immediately transferred to liquid nitrogen. Samples were stored at −80 °C until further processing.

### RNA extraction and cDNA synthesis

The Lipid tissue extraction kit (Qiagen, Hilden, Germany) was utilized to extract RNA from dissected tissues. DNaseI treatment was performed to remove traces of genomic DNA contamination. Concentration of extracted RNA was assessed using a NanoDrop ND-1000 UV-VIS Specro-photometer. Quality of the extracted RNA to be used in the microarray hybridizations was further assessed using the Agilent 2100 Bioanalyzer. In case of cDNA synthesis, equal quantities of RNA were used as template. Synthesis of cDNA was performed using the Superscript III reverse transcriptase kit (Invitrogen, Carlsbad, USA), and random hexamer primers (Invitrogen) and dNTPs (Roche, Basel, Switzerland), following the manufacturer’s protocol.

### Feeding experiment microarray

After two days of normal feeding, individual 5^th^ instar locusts received a single meal of 1.5% agar based artificial diet containing 2.4% Wesson salt mixture, 0.5% linoleic acid, 0.6% cholesterol, 18.8% Vanderzant vitamin mixture, 50% cellulose, 14% dextrin, 8.1% caseine, 2.8% peptone and 2.8% albumin. One group received this meal complemented with plant derived PI (SBBI; Soybean Bowman Birk Inhibitor, and SBTI; Soybean Trypsin Inhibitor: 1% of total protein content; as previously described^13^), while another group received this meal complemented with BSA to achieve equal total protein contents in the diets. 4 hours after ingestion of the meal, midgut and caeca samples were dissected for RNA extraction. Tissues for RNA extraction were dissected in 3 pools of at least 5 individuals for every condition. For confirmation of the microarray results by qPCR, a separate experiment was conducted in a similar setup.

### Labeling of samples

Starting from total RNA, fluorescently labeled cRNA was generated by combining equal quantities of midgut and caeca RNA. Labeling of the samples was performed with the Quick Amp Labeling Kit (Agilent Technologies, Santa Clara, USA), according to the manufacturer’s instructions. In brief, 1 μg of RNA from each sample was with a Cy5 (red) and Cy3 (green) fluorescent dye. mRNA was reverse transcribed to cDNA by using a poly(dT) primer that is coupled to an antisense T7 promotor. The reverse transcriptase also catalyzes synthesis of the second cDNA strand. Prior to cDNA synthesis, a Spike A or Spike B mix was added to all samples to be labeled with Cy3 or Cy5, respectively. These Spike mixes contain polyadenylated transcripts from the adenovirus E1A, that are premixed in various quantities and ratios. They are used as a control for the workflow. In a second step, the second cDNA strand functions as template for the synthesis of cRNA, for which fluorescently labeled dCTPs (Cy3 or Cy5) are provided. The fluorescently labeled samples were subsequently purified by means of the RNeasy Mini Kit (Qiagen), according to the manufacturer’s instructions. For all samples, the yield and activity were higher than the minimum requirements of 825 ng total cRNA and 8 pmol Cy per μg cRNA, as measured with a NanoDrop spectro-photometer.

### Design of the microarray

To reduce the number of transcripts to be analysed, we eliminated all transcripts that were expressed in neither brain- nor gut tissues, based on a self-self hybridisation protocol (Spit *et al.*^20^). From the 48802 unique ESTs available for *L. migratoria*, 12933 were omitted. This allowed the 35869 remaining transcripts to be spotted on a 4 × 44K Custom Gene Expression Microarray slide (Agilent technologies). For each of the resulting unique sequences, the previously determined best probe was spotted in addition to control features. The remaining spots were filled with random doubles to reach the platform maximum of 44K. For the analysis of three pooled control samples and three pooled experimental samples, an n + 2 A-optimal design was chosen (n = 6). This means that 8 hybridizations were performed in a balanced and even design^56^. The data files and platform information have been deposited in the GEO repository of NCBI, and are accessible under the accession number GSE79995.

### Hybridization, scanning and data analysis

Hybridizations were performed at 65 °C for 17 h. Next, the arrays were washed: twice in GE Wash Buffer 1 (Agilent technologies) for 1 min each, in GE Wash Buffer 2 (Agilent technologies) at 37 °C for 1 min, in acetonitrile (Sigma Aldrich, Saint Louis, USA) for 1 min and in Stabilization and Drying Solution (Agilent technologies) for 30 sec. Subsequently, the microarray slides were scanned using a Genepix Personal 4100A confocal scanner (Molecular devices, Sunnyvale, USA) at a resolution of 5 μm and excitation wavelengths of 635 nm and 532 nm. The photomultiplier tube (PMT) voltages for separate wavelengths were adjusted to obtain an overall green/red ratio as close as possible to 1. Images were processed using GenePix Pro 6.0 software (Molecular devices) for spot identification and quantification of the fluorescent signal intensities. The data obtained from the eight hybridizations were used for statistical analysis by means of the R package limma^57^. Spots for which FG < BG + 2SD on all arrays, were deleted before analysis. Median intensities were background corrected using a normal-exponential convolution model and were normalized by Locally Weighed Scatterplot Smoothing (Loess)^58^. For each target transcript a linear model was fitted to the intensity ratios. Next, transcripts were ranked in order of evidence of differential expression using an empirical Bayes method and ‘PI-fed *vs* control’ contrasts were evaluated by the linear models^59^. False discovery rate (FDR) control was obtained by calculating adjusted p-values according to the Benjamini-Hochberg procedure and a cut-off was set at a FDR of p < 0.10^60^. Differentially regulated transcripts were functionally annotated using the InterProScan (IPS) tool that is integrated in the Blast2GO software^25^,^26^. In addition, Gene ontology (GO) annotation was performed using the Blast2GO software^61^. The best blast hit was used for sequence description.

### Transcript assembly and phylogenetic analysis of hexamerin-like proteins

Transcripts putatively encoding hexamerin-like proteins that were identified from an upregulated set of sequences after PI feeding were assembled using DNA Baser software. Transcripts that showed at least 98% nucleotide identity were considered belonging to the same sequence. Signal peptide predictions were made using SignalP4.1^62^ and multiple sequence alignments of insect hexamerin-like proteins were made using MAFFT alignment software^63^. Phylogenetic analysis was performed using MEGA version 6^64^ with aligned amino acid sequences that were manually trimmed to obtain regions with the highest homology. The evolutionary history was inferred by using the Maximum Likelihood method based on the Jones w/freq. model. The bootstrap consensus tree was inferred from 100 replicates. Positions containing gaps were treated as missing data and were eliminated. Protein sequences of hemocyanin from the decapod *Panulirus interruptus* were used to root the tree.

### Synthesis of dsRNA for RNAi studies

Double stranded RNAs for gfp (589 bp), *LmHex2* (715 bp), *LmHex3* (715 bp), *LmHex6* (735 bp) and *LmMet* (421 bp) were synthesized using the MEGAscript RNAi kit (Thermo Fisher Scientific, Waltham, USA), according to the manufacturer’s instructions. Sense and antisense strands were synthesized from a PCR-generated DNA template containing T7 RNA Polymerase promoters on both 5′ ends of the primers. Primer sequences are presented in Supp. Table S8. For *LmHex2* and *LmHex3* the DNA template could be generated in a single PCR reaction using the same primers. cDNA derived from fat body of fifth larval *L. migratoria* was used as a template for the PCR reactions. The PCR reaction was performed using REDTaq ready mix (Sigma) as a source of DNA polymerase, dNTPs and PCR buffer. Amplification of a PCR product of the desired length was checked by performing 1% gel electrophoresis. For the production of gfp dsRNA, an amplified gfp cDNA fragment was cloned in both sense and antisense direction in a TOPO 4.1 sequencing vector (Thermo Fisher Scientific), containing a T7 promotor site. Both RNA strands were then synthesized using T7 enzyme mix (Thermo Fisher Scientific) and sense and antisense strand were annealed subsequently. After dsRNA production, the DNA and ssRNA molecules were removed by nuclease treatment and dsRNA was further purified by solid phase adsorption purification, according to the manufacturer’s instructions. The concentration of the produced dsRNA was estimated using a NanoDrop ND-1000 UV-VIS Specro-photometer, and 1% agarose gel electrophoresis was performed to assess the integrity of the dsRNA.

### Simultaneous knockdown of hexamerin like sequences and PI feeding experiment

At the first day after ecdysis (day 0), 130 5^th^ instar nymphs were weighted and equally divided in two groups. One group was injected with 600 ng from a mixture of dsRNA for *LmHex2*, *LmHex3* and *Lmhex6*, while the other group was injected with 600 ng of dsRNA for gfp as a control. dsRNA was injected directly in the body cavity using a 710RN 100 μl syringe (Hamilton). For three days locusts were fed daily with fresh grass. At day 3 locusts were weighted and both groups of nymphs were again equally subdivided into two groups each, which were transferred to an artificial diet, containing SBBI or SBTI (1%) or BSA, similar to the microarray feeding experiment. Consequently, these divisions resulted in four different treatments: Control-control, control-PI, RNAi-control and RNAi-PI, where the first part of the name refers to the dsRNA treatment and the second to the diet that was received starting from day 3. For the next 4 days locusts received freshly prepared artificial diet daily. At day 7 locusts were weighted and for each condition, midguts and brains were dissected for RNA extraction in 5 pools of at least 5 individuals.

### Met knockdown experiment

At the first day after ecdysis (day 0), a total of 85 5^th^ instar nymphs were divided in two groups. One group was injected with 600 ng of *LmMet* dsRNA, while the other group was injected with 600 ng of dsRNA for gfp as a control. Since initial knockdowns of the receptor proved difficult, locusts received a boost injection at day 2 to maximize the effect. During the experiment, locusts were fed *ad libitum* with grass. On day 4, midguts were collected 3 hours post feed for total RNA extraction. Tissues for RNA extraction were dissected in 6 pools of at least 5 individuals.

### Methoprene treatments

At the first day after ecdysis (day 0), 60 male nymphs were divided in two groups. An experimental group of 30 nymphs was topically treated with the JH III hormone mimic, methoprene (Sigma Aldrich), dissolved in acetone, while a control group received only acetone. The locusts were treated with a daily dose of 100 μg for four consecutive days. During the experiment, locusts were fed with grass *ad libitum*. On day 4, midguts were collected 3 hours post feed for total RNA extraction. Tissues for RNA extraction were dissected in 5 pools of at least 4 individuals.

### RT-qPCR

Quantitative real time RT-PCR was performed according to MIQE requirements^65^. Primer express software (Applied Biosystems) was used to design primers. All primer sequences are displayed as Supp. Table S8. All primer pairs were validated with a standard curve based on a serial dilution of cDNA to determine the primer annealing efficiency. A dissociation protocol was performed to detect the presence of primer dimers and ensure production of a single PCR product. For all tested transcripts, only a single melting peak was found. qPCR reactions using a SYBR green protocol (Invitrogen) were performed in duplicate in 96 well plates according to the manufacturer’s instructions, and were analyzed by the StepOne Plus System (ABI Prism, Applied Biosystems). Relative transcript levels were calculated using the ΔΔCt method^66^. To correct for sample to sample variation, expression was normalized against *rp49* and *rps13*, the two most stably expressed reference genes, as determined with geNorm^67^.

### Statistical analyses

Weight gain and gene expression data were tested for normality by the D’Agostino-Pearson omnibus normality test^68^. In case of weight data, frequency distributions were calculated and non-linear Gaussian regression was used to fit the data. When data were distributed normally, significant differences between two groups were determined by student t-tests. Significant differences for multiple groups were determined by ANOVA. When data were not distributed normally, nonparametric alternatives were used for comparing two or multiple groups, i.e. Mann-Whitney-Wilcoxon (MWW) or Kruskal-Wallis tests, respectively. All statistical analyses were performed using GraphPad Prism software.

### Availability of data and materials

The datasets supporting the conclusions of this article are available in the GEO repository of NCBI and are accessible under the accession number GSE79995, or can be found on the following website http://www.ncbi.nlm.nih.gov/geo/query/acc.cgi?acc=GSE79995. In addition, several datasets supporting the conclusions of this article are included within the article and its additional files.

## Additional Information

**How to cite this article**: Spit, J. *et al.* Transcriptional analysis of the adaptive digestive system of the migratory locust in response to plant defensive protease inhibitors. *Sci. Rep.* **6**, 32460; doi: 10.1038/srep32460 (2016).

## Supplementary Material

Supplementary Information

Supplementary Table S2

## Figures and Tables

**Table 1:** Summary of InterPro families from upregulated transcripts that were annotated in *L. migratoria* after PI ingestion.

Putative function	InterPro§	# Seq¥	Description	Fold Change¤
Storage protein/JH-binding	IPR013788	21	Hemocyanin/hexamerin	1.4–2.8
	IPR010562	2	Haemolymph juvenile hormone binding	1.4–1.5
Protein digestion	IPR001254	16	Serine protease family S1	1.4–2.0
	IPR000834	5	Peptidase M14, carboxypeptidase A	1.3–1.6
Carbohydrate digestion	IPR001360	1	β-glucosidase	1.6
	IPR000933	1	α-L-fucosidases	1.7
	IPR001139	1	Glucosylceramidase	1.5
Lipid digestion	IPR000734	1	Lipase	1.6
Detox/Stress response	IPR001128	3	Cytochrome P450	1.3–2.0
	IPR002018	2	Carboxylesterase, type B	1.4–1.6
Peritrophic membrane	IPR002557	7	Chitin binding domain	2.0–2.1
Other	IPR001071	1	α-tocopherol transport	1.6
	IPR000863	1	Sulfotransferase domain	1.5
	IPR012337	1	Ribonuclease H-like domain	1.4
	IPR001888	2	Transposase/methyltransferase	1.4
	IPR002110	1	Ankyrin repeat	1.4
	IPR011042	1	Six-bladed beta-propeller, TolB-like	1.4
	IPR008037	1	Proteinase inhibitor I19, pacifastin	1.3

^§^InterPro domain ID based on the InterPro database scan^69^.

^¥^Number of transcript sequence occurences corresponding to the indicated InterPro ID.

^¤^When multiple transcripts are identified the range between the minimal and maximal fold change is presented.

**Table 2:** Summary of InterPro families from downregulated transcripts that were annotated in *L. migratoria* after PI ingestion.

Putative function	InterPro§	# Seq¥	Description	Fold Change¤
Structural	IPR004000	15	Actin-related protein	0.6–0.7
	IPR002928	6	Myosin	0.7–0.8
	IPR001781	4	Zinc finger, LIM-type	0.6–0.8
	IPR002035	3	von Willebrand factor, type A	0.4–0.7
	IPR013098	2	Immunoglobulin I-set domain	0.7–0.8
	IPR027707	2	Troponin T	0.6–0.7
	IPR001715	1	Calponin homology domain	0.7
	IPR000301	1	Tetraspanin	0.8
	IPR001152	1	Thymosin β-4	0.8
Defense	IPR001304	3	C-type lectin	0.4–0.6
Carbohydrate metabolism	IPR001360	3	β-glucosidase	0.4–0.6
	IPR001312	2	Hexokinase	0.5–0.7
	IPR001701	1	Cellulase	0.7
Lipid metabolism	IPR000566	1	Lipocalin	0.7
	IPR002172	1	Low-density lipoprotein (LDL) receptor class A	0.8
Other metabolism	IPR002198	1	Short-chain dehydrogenase	0.7
	IPR002085	1	Long-chain alcohol dehydrogenase	0.7
	IPR023186	1	Inosine/uridine-nucleoside hydrolase	0.8
	IPR001148	1	Alpha carbonic anhydrase	0.7
	IPR008597	1	Destabilase	0.6
Protein digestion	IPR000994	1	Peptidase M24, aminopeptidase	0.7
	IPR008257	1	Peptidase M19, dipeptidase	0.6
Detox/Stress response	IPR002018	3	Carboxylesterase, type B	0.6–0.7
	IPR002007	1	Haem peroxidase	0.7
	IPR002403	1	Cytochrome P450, E-class, group IV	0.7
	IPR008978	1	Heat shock HSP20-like chaperone	0.6
Other	IPR002048	1	EF-hand domain	0.7
	IPR011011	1	Zinc finger, FYVE/PHD-type	0.7
	IPR001253	1	Translation initiation factor 1A (eIF-1A)	0.6
	IPR020309	1	Uncharacterized protein family, CD034/YQF4	0.7
	IPR004119	1	Protein of unknown function DUF227	0.5
	IPR012464	1	Protein of unknown function DUF1676	0.6

^§^InterPro domain ID based on the InterPro database scan^69^.

^¥^Number of transcript sequence occurences corresponding to the indicated InterPro ID.

^¤^When multiple transcripts are identified the range between the minimal and maximal fold change is presented.
