# Supplementary material for: Transcriptional Analysis of The Adaptive Digestive System of The Migratory Locust in Response to Plant Defensive Protease Inhibitors
Source: Sci Rep. 2016 Sep 1;6:32460. doi: 10.1038/srep32460 (PMC5007527; doi:10.1038/srep32460)
Supplement: Supplementary Information [file srep32460-s1.pdf]

TRANSCRIPTIONAL ANALYSIS OF THE ADAPTIVE DIGESTIVE SYSTEM OF THE MIGRATORY LOCUST IN RESPONSE TO PLANT DEFENSIVE PROTEASE INHIBITORS

Jornt Spit[1¶], Michiel Holtof [1¶], Liesbet Badisco[1], Lucia Vergauwen[2,3], Elise Vogel[1], Dries Knapen[2,3], Jozef Vanden Broeck[1*]

Supplementary Figure S1. Comparison of fold changes observed in the micro-array analysis with RT-qPCR results. The relative quantity (RQ) of transcripts was normalized against two reference genes, *rp49* and *rps13*, and calibrated to control = 1, dotted black line. The observed microarray fold change is represented by a striped red line. Means ± SEM are presented for each transcript tested (n = 3 pools, ≥ 4 individuals per pool).

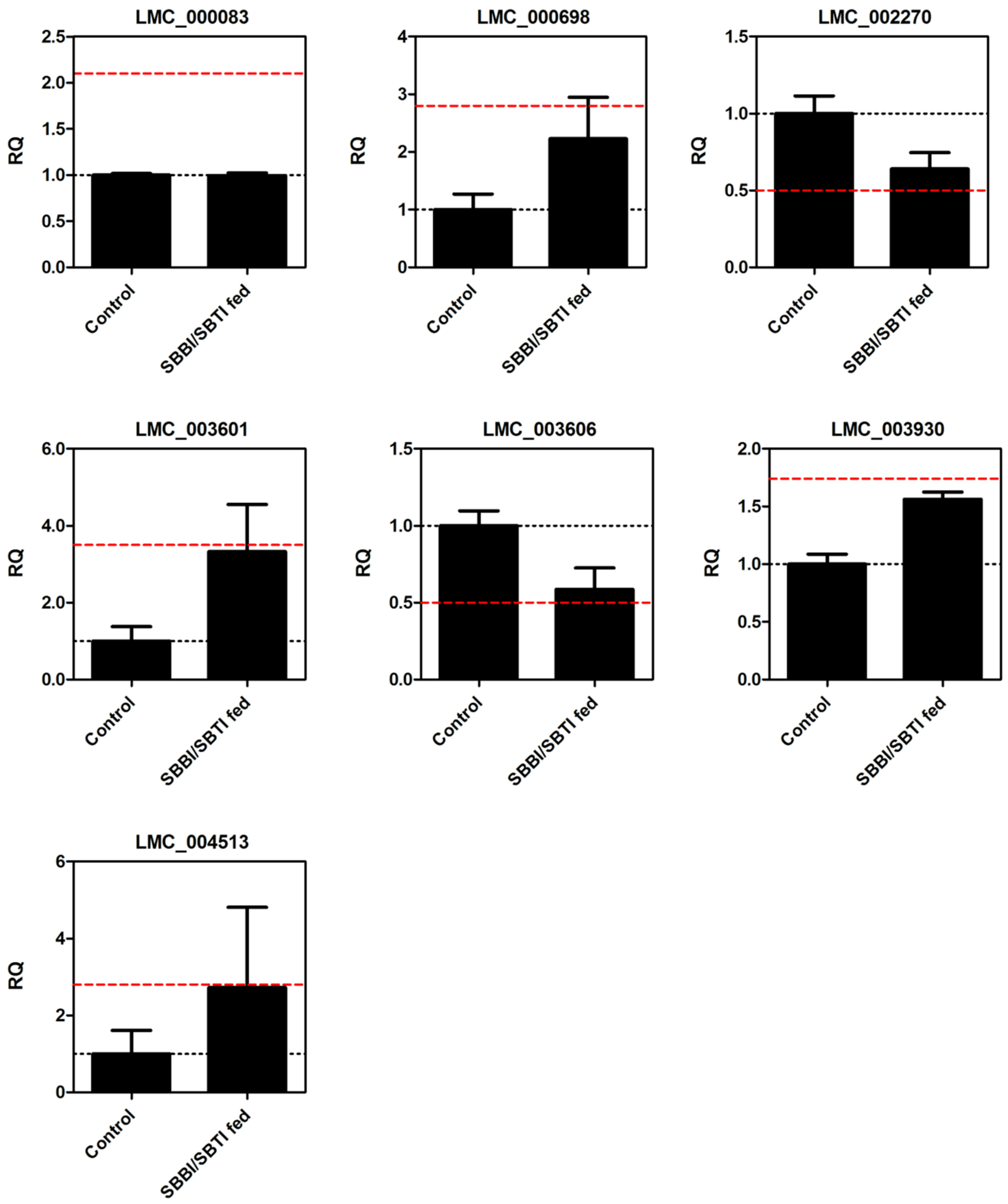

Supplementary Figure S3. DNA sequence and deduced ORF from *Locusta migratoria* hexamerin-like protein 2, 3 and the newly identified *LmHex6*. Underlined amino acids represent a signal peptide with an arrowhead indicating the putative cleavage site. Arrows above the DNA sequence indicate primer positions used for RT-qPCR, while nucleic acid residues shaded in grey represent the fragment amplified for RT-qPCR detection. Arrows below DNA indicate primer sequences used for the construction of a dsRNA fragment. Underlined and italic nucleic acid residues correspond to the fragment that is amplified for the transcription of the dsRNA. Amino acids shaded in black are signature motifs from insect hexamerin like proteins.

LmHexamerin-like protein 2

```
ATCCCCGGGCTGCAGAATTCGCAAGCGAGCAACATGAGGACTGCGACCGTTGTCGTCCTGTCACTGTTGGCAGCCCTTGCGGCCGCCGCG
                                             M  R  T  A  T  V  V  V  L  S  L  L  A  A  L  A  A  A  A▼
GTTGTACCGCACAGCGAGGCCGGTAAGGAACTTCTGGAGAAACAAGACAAGCTTCTGCGGCTCTTGTACCATGTACAACAGCCAACACTC
 V  V  P  H  S  E  A  G  K  E  L  L  E  K  Q  D  K  L  L  R  L  L  Y  H  V  Q  Q  P  T  L
GTCAAAGAAGAGCAGGAAATAGCCAAGACCTACAAGCCTATTGAGCATGTCGACAACTACCAGTACAAAGACAAGGTAGAACTCTTCTGG
 V  K  E  E  Q  E  I  A  K  T  Y  K  P  I  E  H  V  D  N  Y  Q  Y  K  D  K  V  E  L  F  W
AAGTATTACGTCGATGTTGGCTTCCTGCCGAAAGGAGAAGTTTTCTCTGTTTACTACCAGAAGCACTTTTACCAAGCCCGGGCATTGTTT
 K  Y  Y  V  D  V  G  F  L  P  K  G  E  V  F  S  V  Y  Y  Q  K  H  F  Y  Q  A  R  A  L  F
GAGCTGTTCTTCTTCGCCAAGGACTTCGAGACGTTCTACAAGACCGCCGTTTGGGCCCGGGAGCACCTGAACGAGGCGCTGTTCGTCTAC
 E  L  F  F  F  A  K  D  F  E  T  F  Y  K  T  A  V  W  A  R  E  H  L  N  E  A  L  F  V  Y
TCATACACCGTCGCTGTGCTGCATCGCGAGGACACCAAGGACGTGACGCTGCCAGCTCCCTACGAGGTCTACCCGCAGCTGTTCGTCAAC
 S  Y  T  V  A  V  L  H  R  E  D  T  K  D  V  T  L  P  A  P  Y  E  V  Y  P  Q  L  F  V  N
GCAGAGGTCATCCAGCAGGCGTACGACGCCTACCTGAGAGGTGAGGTCGGCACCAAGGAAGCCCCTTACGTGTTCTACTCCAACTACAGC
 A  E  V  I  Q  Q  A  Y  D  A  Y  L  R  G  E  V  G  T  K  E  A  P  Y  V  F  Y  S  N  Y  S
GGCTACCCCGTGGCGAGCAACCCTGAAGAGCTGGTTTCCTACTTTACCGAAGACATTGGCCTCAACTCGTACTTCGCGTACCTGAGCTAC
 G  Y  P  V  A  S  N  P  E  E  L  V  S  Y  F  T  E  D  I  G  L  N  S  Y  F  A  Y  L  S  Y
AAGTACCCATACTGGCTCAACCCCAAGAACTACAGCCTTCCCGAGTACCAGTACCGTGGAGAGAGCTTCTTCTTCGTCCTCCAACAACTG
 K  Y  P  Y  W  L  N  P  K  N  Y  S  L  P  E  Y  Q  Y  R  G  E  S  F  F  F  V  L  Q  Q  L
CTGGCTCGCTACTACCTGGAGAGGCTGTCCAATCACCTTCCTGACGTAAAGGCTGTCGACTACAACCACCCAGTCCTGGTCGGCTACTAC
 L  A  R  Y  Y  L  E  R  L  S  N  H  L  P  D  V  K  A  V  D  Y  N  H  P  V  L  V  G  Y  Y
CCTGAGCTGAGACTGCAGAATGGACGTGAGGCTCCCGCGCGACCTGAGGGAATCTTCGCCCGAAACGTCGACATCCTGTACGTCGAGGAG
 P  E  L  R  L  Q  N  G  R  E  A  P  A  R  P  E  G  I  F  A  R  N  V  D  I  L  Y  V  E  E
ATCAGGAACTACGAGAGGAGGATCCGCGACGGAATTGACTACGGCTACCTGGCAGGCTACAACTACGAGAAGTACAACGTGAGGGAGAAG
 I  R  N  Y  E  R  R  I  R  D  G  I  D  Y  G  Y  L  A  G  Y  N  Y  E  K  Y  N  V  R  E  K
GACTACACCAACGTCCTTGGCAATATCCTAGAGGGCAATGAGGACTCCATCAACAAGGAATACTACGGCGCTTTCTACAGGAACCTCATC
 D  Y  T  N  V  L  G  N  I  L  E  G  N  E  D  S  I  N  K  E  Y  Y  G  A  F  Y  R  N  L  I
TCCCTATTTGGTCACATTGTCGATCCTGTCCACCGATATGGAGTTCCTGCTAGCGTTCTCGAACAGCCCGAAACCCAGCTGAGGGACCCC
 S  L  F  G  H  I  V  D  P  V  H  R  Y  G  V  P  A  S  V  L  E  Q  P  E  T  Q  L  R  D  P
CTGTTCTACAGGATTGGGAAGCGCGTTCTGTCCATCTTCTACCACTACAAGAACCTTCTGAGGCCCTACACTCATGAAGATCTGTACCTG
 L  F  Y  R  I  G  K  R  V  L  S  I  F  Y  H  Y  K  N  L  L  R  P  Y  T  H  E  D  L  Y  L
CCAGGCGTCACCGTGGAAGACATCACCTTCGACAAGCTCGTCACCTTCTTCGACACCTTCGACTTCGAAATCAACAACGCCCTGACCTTA
 P  G  V  T  V  E  D  I  T  F  D  K  L  V  T  F  F  D  T  F  D  F  E  I  N  N  A  L  T  L
TCCAAGCCTGAAGAGGGCGCTGGGTTCAGCTACGTCGCTCGCCAGTACCGCCTGAACCACAAGCCGTTCTTCTACCACCTCAAAGTTAAG
 S  K  P  E  E  G  A  G  F  S  Y  V  A  R  Q  Y  R  L  N  H  K  P  F  F  Y  H  L  K  V  K
AGCGAGAAGGAAGTCGACTCCGTCGTCCGAGTCTTCATTGGTCCCAAATACGACGCCCTCGGCCGCGAATACAGCCTGGAGGAGAGGAAG
 S  E  K  E  V  D  S  V  V  R  V  F  I  G  P  K  Y  D  A  L  G  R  E  Y  S  L  E  E  R  K
CAGTACTACGTTCTGCTGGACACCTTCAACTATAAGCTGGTCGCTGGTGAGAACGACATCAAGCGCAGCTCCAACGACTTCCCGTTCTAC
 Q  Y  Y  V  L  L  D  T  F  N  Y  K  L  V  A  G  E  N  D  I  K  R  S  S  N  D  F  P  F  Y
GCCAAGGAGGCGCCCAGCTGGTACGACCTCTACAAGGCCACGAGCAGCGCCGTCAAGGGCGAGGACAAGTTCTTCCTGGACAAGTTCCGC
 A  K  E  A  P  S  W  Y  D  L  Y  K  A  T  S  S  A  V  K  G  E  D  K  F  F  L  D  K  F  R
TCGCACTTCGGCTTCCCGCAGAGGCTGGCGCTGCCCCGCGGTACCCGCAGTGGTCTGCCACTCAGCGTGTTCACCATCGTCACGCAAGCC
 S  H  F  G  F  P  Q  R  L  A  L  P  R  G  T  R  S  G  L  P  L  S  V  F  T  I  V  T  Q  A
TCCCCTGACGCCAAGAACCCCATCCTGGAGCACGGAGACCTGCACGCCGCCGGCTTCCCGTTCGACAGGCGCGTCGTCGAGTTCGAGTTC
 S  P  D  A  K  N  P  I  L  E  H  G  D  L  H  A  A  G  F  P  F  D  R  R  V  V  E  F  E  F
GACGTGCCCAACGCGCACTTCGACGAGACGTTCGTCGTGCACCGCCGCGTGGAGGACCTCAACGCCACGGCCTGAACTGCCCATGTTCCT
 D  V  P  N  A  H  F  D  E  T  F  V  V  H  R  R  V  E  D  L  N  A  T  A  -
CGAACATCACCATCAGACTGACCTACTTGCTCATCAACTCGCGATCACTTTTTTAAAAGCCTTTTTTACTTTTTCGCAAAAAATAAAAAT
TGGAAAGTAAAAAAAAAAAAAAAAAAAAAAAAA
```

LmHexamerin-like protein 6

```
CCCCGGGCTGCAGGCACGAGCAACATGAGGACTGCAACCGTGGTCGTCTTGTCGCTGTTGGCTGCCCTGGCGGCGGCCACCGCAGTACCG
                          M  R  T  A  T  V  V  V  L  S  L  L  A  A  L  A  A  A  T  A  V ▼P
CCCAGTGAGGCTGACAAGGAACTTCTGGAGAAACAGAACAAGATTATAAGGCTTTTCTACCAAGTACAACAACCAACAATTATCCCAGAA
 P  S  E  A  D  K  E  L  L  E  K  Q  N  K  I  I  R  L  F  Y  Q  V  Q  Q  P  T  I  I  P  E
GAACAAGAAATAGCCAAGTCCTACAAGCCAATTGAGAATATCGACAACTACCAGTACAAGGACAAAGTCGAAGTCTTCTGGAAGTACTAC
 E  Q  E  I  A  K  S  Y  K  P  I  E  N  I  D  N  Y  Q  Y  K  D  K  V  E  V  F  W  K  Y  Y
ACGGAGTACGGGTTCGTGCCCCGAGATGAAGTGTTCTCGATCTACTACAAGAAACACTTCTCCCAGGCGAAAGGCCTGTTCGAGCTGTTC
 T  E  Y  G  F  V  P  R  D  E  V  F  S  I  Y  Y  K  K  H  F  S  Q  A  K  G  L  F  E  L  F
TACTACGCCAAGGACTTCGACACCTTCTACAAGACGGCTGTTTGGGCGAGAGAATACTTGAACCCAGGACTGTTCGTGTACTCATTCACT
 Y  Y  A  K  D  F  D  T  F  Y  K  T  A  V  W  A  R  E  Y  L  N  P  G  L  F  V  Y  S  F  T
GTCGCTGTGCTGCATCGCGAGGATACCAAGTTCGTCACCCTGCCAGCTCCCTATGAAGTCTACCCACAACTCTTCGTCAACGCCGAGGTC
 V  A  V  L  H  R  E  D  T  K  F  V  T  L  P  A  P  Y  E  V  Y  P  Q  L  F  V  N  A  E  V
ATCCAGAAGGCTTACGACGCACGCCTGAGAGATGTCGTCAGCACCAGGAAGGAGCCGTACGTGTTCTACGCCAACTACAGCGGCTTCCCC
 I  Q  K  A  Y  D  A  R  L  R  D  V  V  S  T  R  K  E  P  Y  V  F  Y  A  N  Y  S  G  F  P
GTCGCCAACAACCCCGAGGAGCTGGTGTCGTACTTCACCGAGGACGTCGGGCTGAACTCGTTCTTCGCCTACCTGCACTACAAGAGCCCC
 V  A  N  N  P  E  E  L  V  S  Y  F  T  E  D  V  G  L  N  S  F  F  A  Y  L  H  Y  K  S  P
TTCTGGCTGAACCCGGCCAACTACAGCCTGCCCGCCTCCAAGCGCCGCGGAGACAGCTTCTTCTTCATCCTGCAGCAGCTGCTGGCCCGC
 F  W  L  N  P  A  N  Y  S  L  P  A  S  K  R  R  G  D  S  F  F  F  I  L  Q  Q  L  L  A  R
TACTACCTGGAGAGGCTGTCCAACCGCCTGCCCGACGTCAAGCCCGTCGACTACGCCAACCCCGTGCTGGTGGGCTACTACCCAGAGCTG
 Y  Y  L  E  R  L  S  N  R  L  P  D  V  K  P  V  D  Y  A  N  P  V  L  V  G  Y  Y  P  E  L
AGGCTGCAGAACGGCATCGAGGCCCCCGCCCGCCCTGAAGGCGTCTACCCCAGCAACTTCGACCTGCTGTTCGTGGAGCGCATCCAGAAC
 R  L  Q  N  G  I  E  A  P  A  R  P  E  G  V  Y  P  S  N  F  D  L  L  F  V  E  R  I  Q  N
TACGAGAGGAGGATCAGGGACGCTGTTGACTTTGGCTACCTATATGGCTATGACTTCAAGACATTCAACCTGAACGAGAAGGACCTGACC
 Y  E  R  R  I  R  D  A  V  D  F  G  Y  L  Y  G  Y  D  F  K  T  F  N  L  N  E  K  D  L  T
GACATCCTTGGAAACGTCATTGAGGGCAACGCCGAATCCGTCAACTACGAGTTCTACGGCTCCATCTACCGGTACCTCATCTCCCTCTTC
 D  I  L  G  N  V  I  E  G  N  A  E  S  V  N  Y  E  F  Y  G  S  I  Y  R  Y  L  I  S  L  F
GGCCACATTGCTGACCCGTACCACAAGTACGGCGCACCTGCCAGTGTTCTTGAGCAGCCAGAGACACAGTTGAGGGACCCCCTGTTCTAC
 G  H  I  A  D  P  Y  H  K  Y  G  A  P  A  S  V  L  E  Q  P  E  T  Q  L  R  D  P  L  F  Y
AGAATCGCCAAGCGAGTCATTTCCATCTTCTACCAGTACAAGAACCAGCTGAAGCCATACACCAAGAACCAACTGGAATTCCCTGGTGTC
 R  I  A  K  R  V  I  S  I  F  Y  Q  Y  K  N  Q  L  K  P  Y  T  K  N  Q  L  E  F  P  G  V
GCTATTGAGGGTATCACCTTCGACAAGCTCGTCACGTTCTTCGACGACTTTGACATCGAGCTGAACAATGCCCTGTCCTTCTCAAAGCCC
 A  I  E  G  I  T  F  D  K  L  V  T  F  F  D  D  F  D  I  E  L  N  N  A  L  S  F  S  K  P
GAACAAGGTGACAACTTCAACTTCATTGCCCGCCAGTACCGCCTGAACCACAAGCCCTTCTACTACCAGCTGAAGGTGAAGAGCGAGAAG
 E  Q  G  D  N  F  N  F  I  A  R  Q  Y  R  L  N  H  K  P  F  Y  Y  Q  L  K  V  K  S  E  K
GAAGTCGACGCTGTAGTGCGCGTCTTCGTTGGACCCAAGTACGACGTGTACGGCCGCGAATTCACACTGGACGAGAAGAAGCAGTACTAC
 E  V  D  A  V  V  R  V  F  V  G  P  K  Y  D  V  Y  G  R  E  F  T  L  D  E  K  K  Q  Y  Y
TTCCTGCTGGACGTCTTCAACCAGAAACTGAACGCCGGCGAGAACGAGATCAAGCGCAGCTCCAAGGAGTTCGCGTTGTTCGCCAAGGAG
 F  L  L  D  V  F  N  Q  K  L  N  A  G  E  N  E  I  K  R  S  S  K  E  F  A  L  F  A  K  E
GCGCCCAGCTACTACGACCTGTACCAGACGACGTACCGCGCCCTCAAGGGCGAGGACAAGTTCTCCCTGGACAAGTTCCGCTCGCACTTC
 A  P  S  Y  Y  D  L  Y  Q  T  T  Y  R  A  L  K  G  E  D  K  F  S  L  D  K  F  R  S  H  F
GGCTTCCCTCAGCGGCTGGCGCTGCCGCGCGGTACCCGCAGCGGTCTGCCACTCAGCGTGTTCGCCATCGTCACCCCCGCCGTGCAGGGA
 G  F  P  Q  R  L  A  L  P  R  G  T  R  S  G  L  P  L  S  V  F  A  I  V  T  P  A  V  Q  G
TCCGAGCACCCCGTGCTGCCGTACTACGACAACCAGGCCGCGGGATTCCCGTTCGACAGGCGCGTCGTCGAGTTCGAGTTCGACGTGCCC
 S  E  H  P  V  L  P  Y  Y  D  N  Q  A  A  G  F  P  F  D  R  R  V  V  E  F  E  F  D  V  P
AACGTGTACTTCGGCGAGACGTACGTCGTGCACCGCCGCGTCGAGGACATCAACACCACCGCCTGAATGGCTCCAGTGCCACCGTCGCAC
 N  V  Y  F  G  E  T  Y  V  V  H  R  R  V  E  D  I  N  T  T  A  -
TGGCGGCGCTGCTTGTGGAACTCCACCTCCACTGTACTGTGTCCTCACATACAATAAAGAGCTGATGCTTTAAAAAAAAACAAA
```

LmHexamerin-like protein 3

TCCCCAGGCGAACCAACCGACTCCAGCAACATGAGGACTGCGACCGTTGTCGTCCTGTCACTGTTGGCAGCCCTTGCGGCCGCCGCGGTT
M R T A T V V V L S L L A A L A A A A ▼V
GTACCGCACAGCGAGGCCGGTAAGGAACTTCTGGAGAAACAAGACAAGCTTCTGAGGCTCTTGTACCATGTACAACAGCCAACACTCGTC
V P H S E A G K E L L E K Q D K L L R L L Y H V Q Q P T L V
AAAGAAGAGCAGGAAATAGCCAAGACCTACAAGCCTATTGAGAATGTCGACAACTACCAGTACAAAGAAAAAGTAGAACTCTTCTGGAAG
K E E Q E I A K T Y K P I E N V D N Y Q Y K E K V E L F W K
TATTACGTCGATGTTGGCTTCCTGCCCAAAGGAGAAGTTTTCTCTGTTTTCTACCAGAAGCACTTTTACCAAGCCCGAGCATTGTTTGAG
Y Y V D V G F L P K G E V F S V F Y Q K H F Y Q A R A L F E
CTGTTCTACTTCGCCAAAGACTTCGAGACGTTCTACAAGACCGCCGTTTGGGCCCGGGAGCACCTGAACGAGGCGCTGTTCGTCTACTCA
L F Y F A K D F E T F Y K T A V W A R E H L N E A L F V Y S
TACACCGTCGCTGTGCTGCATCGCGAGGACACCAAGGACGTGACGCTGCCAGCTCCCTACGAGGTCTACCCGCAGCTGTTCGTCAACGCA
Y T V A V L H R E D T K D V T L P A P Y E V Y P Q L F V N A
GAGGTCATCCAGCAGGCGTACGACGCCTACCTGAGAAGTGAAGTCGGCACCAAGGAAGCCCCTTATGTATTCTACTCCAACTACAGCGGC
E V I Q Q A Y D A Y L R S E V G T K E A P Y V F Y S N Y S G
TACCCCGTGGCGAGCAACCCTGAAGAGCTGGTTTCCTACTTTACCGAAGACGTTGGCCTCAACTCGTACTTCGCGTACCTGAGCTACAAG
Y P V A S N P E E L V S Y F T E D V G L N S Y F A Y L S Y K
TACCCATACTGGCTCAACCCCAAGAACTACAGCCTTCCCGAGTACAAGTACCGTGGCGAGAGCTTCTTCTTTGTCCTCCAACAACTGCTG
Y P Y W L N P K N Y S L P E Y K Y R G E S F F F V L Q Q L L
GCTCGCTACTACCTGGAGAGGCTGTCCAACCACCTTCCTGACGTCAAGGCTATCGACTACAACCATCCAGTCCTGGTCGGCTACTACCCT
A R Y Y L E R L S N H L P D V K A I D Y N H P V L V G Y Y P
GAGCTGAGACTGCAGAATGGACGTGAGGCTCCAGCGCGACCTGAGGGAATCTTCGCCCGCAACGTCGACATCCTGTACGTCGAGGAAATC
E L R L Q N G R E A P A R P E G I F A R N V D I L Y V E E I
AAGAACTACGAGAGGAGGATCCGCGACGGAATTGACTACGGCTACCTGGCAGGCTACAACTACGAGAAGTACAACGTGAGGGAGAAGGAC
K N Y E R R I R D G I D Y G Y L A G Y N Y E K Y N V R E K D
TACACCAACGTCCTTGGCAACATCCTTGAGGGCAACGACGAGTCCATCAACAAGGAGTTCTACGGCGCTCTCTTCAGGAACCTCATCTCT
Y T N V L G N I L E G N D E S I N K E F Y G A L F R N L I S
CTCTTCGGCCACATTGTCGACCCTGTCCA*CCGATATGGAGTTCCTGCTAGCGTTCTCGAACAGCCCGAAACCCAGCTGAGGGACCCTCTG*
L F G H I V D P V H R Y G V P A S V L E Q P E T Q L R D P L
TTCTACAGTATTGCCAAGCGCGTTTTGTCCATCTTCTACCACTACAAGAACCTCCTGAAGCCTTACACTTATGAGGATTTGTACCTGCCA
F Y S I A K R V L S I F Y H Y K N L L K P Y T Y E D L Y L P
GGCGTCACCGTCGATGACATCACCTTCGACAAGCTCGTCACGTACTTCGACAACTTCGACTTTGAAATCAACAACGCCCTGACCATCTCC
G V T V D D I T F D K L V T Y F D N F D F E I N N A L T I S
AAGCCTGAAGAGGGCGCTGAATTCAGCTACGTCGCTCGCCAGTACCGCCTGAACCACAAGCCGTTCTTCTACCACCTCAAAGTGAAGAGC
K P E E G A E F S Y V A R Q Y R L N H K P F F Y H L K V K S
GAGAAGGAAGTCGACTCCGTCGTCCGAGTCTTCATTGGTCCCAAATACGACGCCCTCGGCCGCGAACTCAGCCTGGAGGAGAGGAAGCAG
E K E V D S V V R V F I G P K Y D A L G R E L S L E E R K Q
TACTACGTTCTGCTGGACACCTTCAACTACAAGTTGGTCGCCGGTGAGAACGACATCAAGCGCAGCTCCATCGACTTCCCGCTCTACGCC
Y Y V L L D T F N Y K L V A G E N D I K R S S I D F P L Y A
AAGGAGGCGCCCAGCTGGTACGACCTCTACAAGGCCACCAGCAGCGCCGTCAAGGGCGAGGACAAGTTCTTCCTGGACAAGTTCCGCTCG
K E A P S W Y D L Y K A T S S A V K G E D K F F L D K F R S
CACTTCGGCTTCCCGCAGAGGCTGGCGCTGCCCCGCGGTACCCGCAGTGGTCTGCCACTCAGCGTGTTCACCATCGTCACGCAAGCCTCC
H F G F P Q R L A L P R G T R S G L P L S V F T I V T Q A S
*CCTGACGCCAAGAACCCCATCCTGG*AGCACGGAGACCTGCACGCCGCCGGCTTCCCGTTCGACAGGCGCGTCGTCGAGTTCGAGTTCAAC
P D A K N P I L E H G D L H A A G F P F D R R V V E F E F N
GTGCCCAACGCGCACTTCGACGAGACGTTCGTCGTGCACCGCCGCGCGGAGGACATCAACGCCACGGCCTGAACTGCCCAAGTTCCTCCA
V P N A H F D E T F V V H R R A E D I N A T A -
ACATCACCATCAGACTGACCCACTTGCCCATCAACTCGCGATCACGTTTTTACAAGCCTTTTTTACTTTTTCGCAAAAAATAAAA

- Stop codon, amino acids shaded in black are signature motifs, underlined amino acids and an arrowhead represent a signal peptide and putative cleavage site, respectively. Arrows above DNA represent primerpairs used for RT-qPCR, while arrows below DNA represent primerpairs used for the construction of dsRNA. Nucleic acid residues shaded in grey represent the fragment amplificated for RT-qPCR detection. Underlined and italic nucleic acid residues correspond to the fragment that is amplificated for the transcription of a dsRNA fragment.

Supplementary Table S4. Percent AA sequence identity table for *L. migratoria* hexamerin-like proteins. LmHex2, LmHex3, and LmHex6 share the most similarity, while LmHex4 and LmJHBP show less resemblance.

	**LmHex2**	**LmHex3**	**LmHex4**	**LmHex6**	**LmJHBP**
**LmHex1**	40.4	44.5	38.4	45.1	37.3
**LmHex2**		95.7	39.6	74.6	40.4
**LmHex3**			39.6	74.7	39.9
**LmHex4**				41.0	35.3
**LmHex6**					42.5

Supplementary Figure S5. Adult tissue distribution of transcript levels for three *L. migratoria* hexamerin-like proteins. Relative quantity (RQ) in eleven adult tissues was examined. Mtb (Malpighian tubules), Br (brain including optic lobes), CA (corpora allata), CC (corpora cardiaca), Fg (foregut), Mg (midgut), Hg (hindgut), Ca (caeca), Fb (fat body), Mu (muscles), Rs (male and female reproductive system). Relative transcript levels are normalized against two reference genes, *rp49* and *rps13*. Y-axes are scaled logarithmically and each horizontal dotted line represents a 10 fold difference in expression. Expression levels between different genes cannot be compared. Boxplots of the results are presented based on 4 pools, 5 individuals per pool.

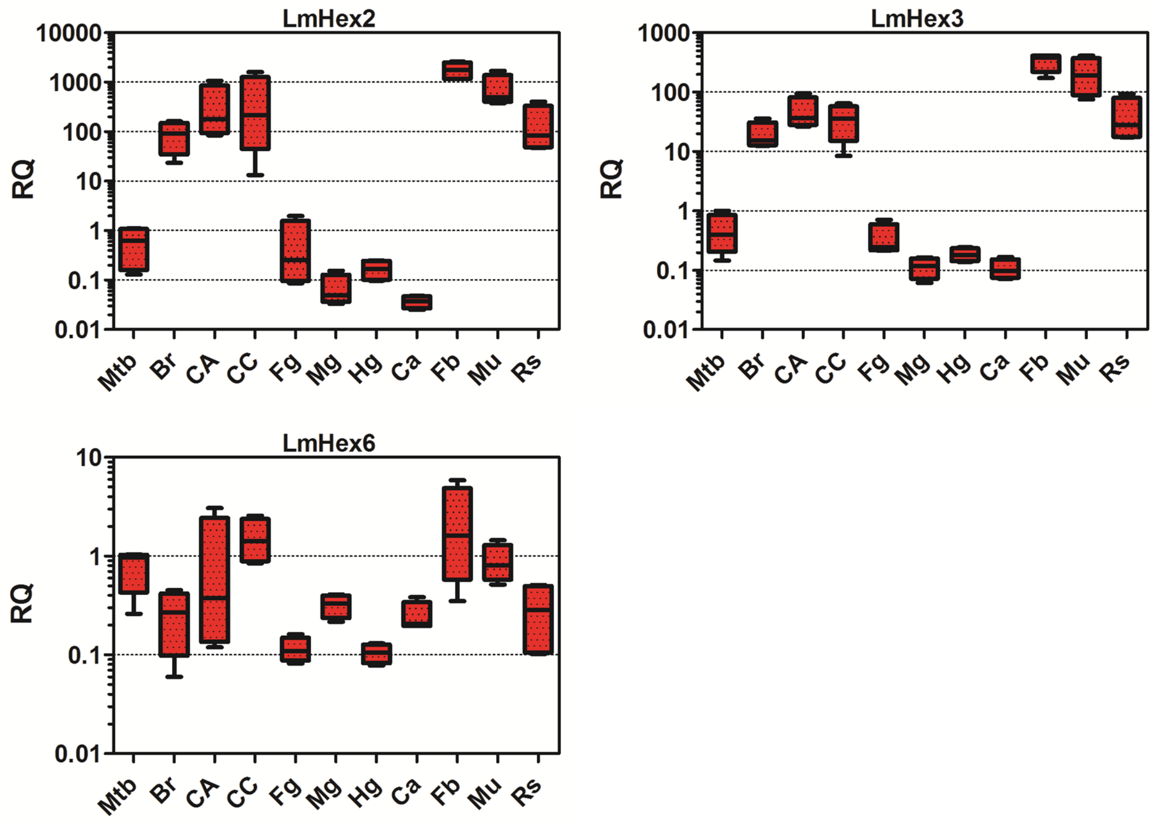

Supplementary Figure S6. Knockdown levels of *LmMet* in brain and midgut of larvae treated with dsRNA targeting the methoprene tolerant receptor. Relative transcript (RQ) levels for *LmMet* are normalized against two reference genes, *rp49* and *rps13* (n > 6 pools, ≥ 4 individuals per pool).

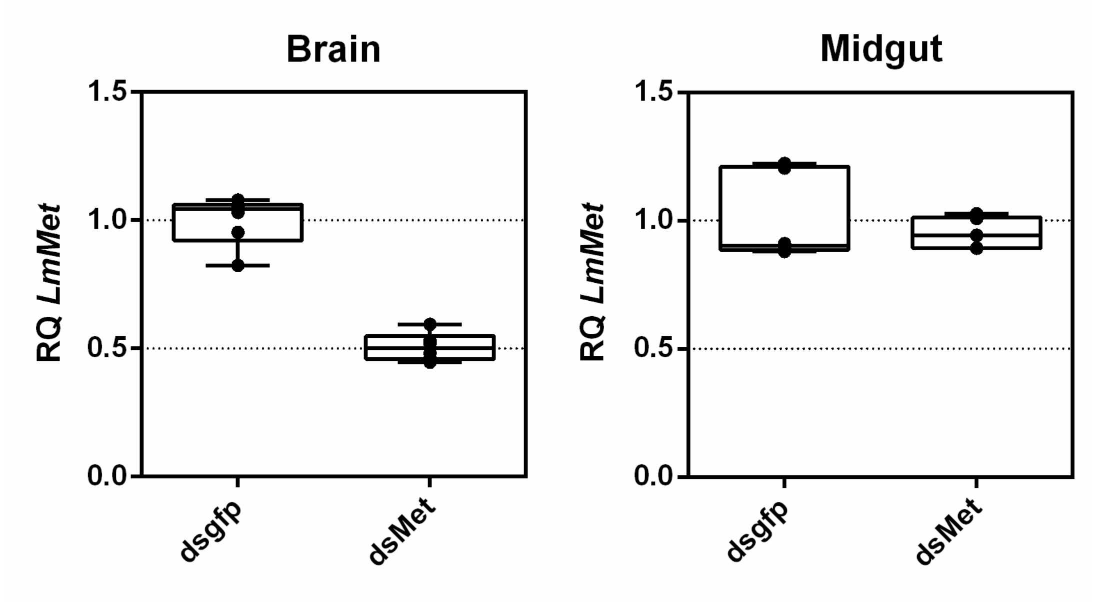

Supplementary Figure S7 Relative transcript levels in the midgut of *LmTry1A*, *LmTry1b*, *LmTry2A*, *LmHex2*, *LmHex3*, and *LmHex6* after knockdown of LmMet (A) or after topical application of methoprene (B). Relative transcript (RQ) levels for *LmMet* are normalized against two reference genes, *rp49* and *rps13*. Boxplots of the results are presented based on 6 pools, at least 4 individuals per pool.

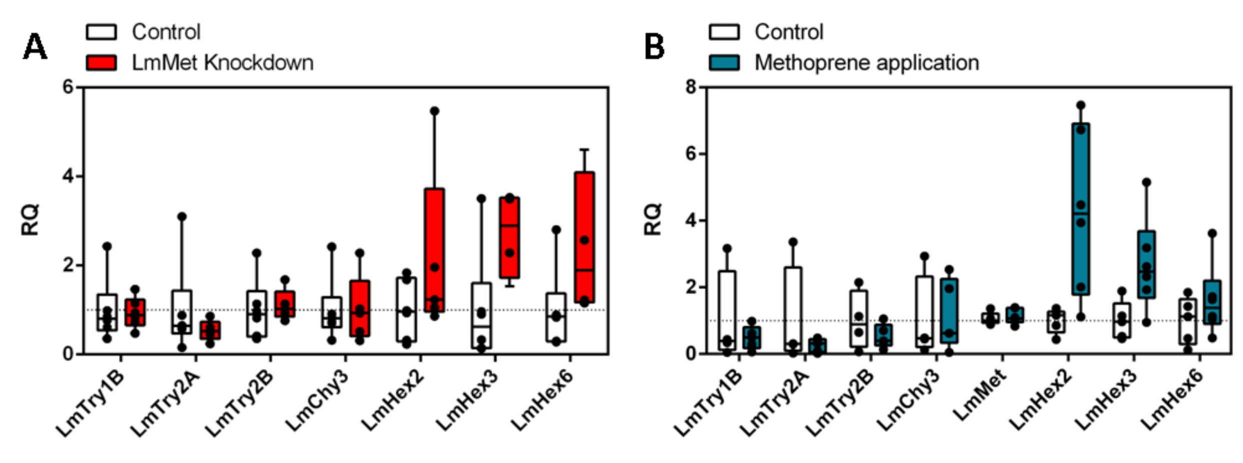

Supplementary Table S8. Overview of primer sequences used throughout this work. Table containing all primer sequences used for the generation of dsRNA fragments, and all sequences used in the RT-qPCR analyses.

Function	Gene-ID	Forward primer sequence	Reverse primer sequence
RT-qPCR	Rp49	CGCTACAAGAAGCTTAAGAGATCATG	CTTAAACCTACGGCGAACTCTGTT
	Rps13	CGTGAAGGGTGATATTTTGCA	GCTGACTGGGATATACCCTTACCAG
	LMS_000083	ACCAGTGTACCTTTGTGAAATCCA	CGCCTGTTCAGCACACCAT
	LMS_000698	ACTTCCTGCTGGACGTCTTCA	TTGGAGCTGCGCTTGATCT
	LMC_002270	GAGAGCATCTGGGACCACATG	GTCACCGTTCTTGCCGTCTAC
	LMC_003601	TGGTTCCAGGTACTTTCAAGAGTCT	TCCGTGTCCTCCTGTCATTTT
	LMC_003606	TGTCTCCAAGTTCCCGAACAT	TCTTCATACGTCCATACTGGTTCAC
	LMC_003930	GACGAAGGTCCTGGATCAGATC	GGCGATGTCGTTAGGGTAGATG
	LMC_004513	GCTCCATCTACCGGTATCTCATCT	GGCAGGTGCGCCGTACT
	LmChy1	GCCAGCTCCGGCTACAAG	CACCTGTACGACGGCAATGT
	LmChy2	TGGCAGCTGTCCTTCCAGTA	ACCCAGTCGGAGCTGATGAT
	LmChy3	ACGGCGACTTCGATACCTACTC	CCACGGCCTGAATATTGTCA
	LmChy4	ACCCAAACCGACATAGCAGAGT	ACGCCCCGCACATGTG
	LmTry1B	CAGTGGCAACGACTACGACATC	CACGTTGGTACCGAAGCTGAA
	LmTry2A	TCGAGGGCTCCTACATCAACTAC	TGCATGACACCGATGTCGTA
	LmTry2B	GACGTGGCCACCATCATTG	CCGTCTATCTCAAGGAGTGCAA
	LmMet	TTAGGGCAGCATCAGAAAG	TCGTCGGGAGGAAGTGTAT
	LmHex2	CCTATTTGGTCACATTGTCGATCC	GCTGGGTTTCGGGCTGTT
	LmHex3	CATCTCTCTCTTCGGCCACATT	GCTGTTCGAGAACGCTAGCA
	LmHex6	GCTCCATCTACCGGTACCTCATCT	GGCAGGTGCGCCGTACT
dsRNA§	LmHex2/3	TAATACGACTCACTATAGGGCCGATAT GGAGTTCCTGC	TAATACGACTCACTATAGGGCCAGGATG GGGTTCTTG
	LmHex6	TAATACGACTCACTATAGGGAAGTACG GCGCACCTG	TAATACGACTCACTATAGGGCCTGGTTG TCGTAG
	LmMet	TAATACGACTCACTATAGGGTTAGGGC AGCATCAGAAAG	TAATACGACTCACTATAGGGTCGTCGGG AGGAAGTGTAT

§ Underlined sequences are T7 promotor sequences necessary for generation of dsRNA from the amplified cDNA fragment
